# Supplementary material for: Music Festival Makes Hedgehogs Move: How Individuals Cope Behaviorally in Response to Human-Induced Stressors
Source: Animals (Basel). 2019 Jul 18;9(7):455. doi: 10.3390/ani9070455 (PMC6680799; doi:10.3390/ani9070455)
Supplement: Supplementary file 1 [file animals-09-00455-s001.pdf]

Table S1. Predicted differences from the fitted models. KDE50, resting, locomotion, balling up, other, and DFC are on their original scale. The KDE50 is measured in hectares, the behaviour variables are counts and the DFC is a proportion. Diurnality was transformed by adding 1 than calculating the natural logarithm. Calculation the exponential of these differences the results represents the differences as a proportion. TSdusk was transformed by adding the absolute value of the lowest TSdusk value to all TSdusk values to get only positive values. The differences still represent the difference in hours. The ODBA was transformed using the z-score. The differences represent changes in the magnitudes of standard deviations.

Parameter	ID	Prediction	95% CI Lower	95% CI Upper
KDE50	01_2016	−0.9	−1.17	−0.74
	02_2016	−0.12	−0.34	0.10
	08_2016	−0.91	−1.13	−0.68
	09_2016	−1.44	−1.77	−1.10
	13_2016	−0.06	−0.28	0.16
	17_2016	−0.86	−1.09	−0.63
	19_2016	−1.73	−1.95	−1.51
	21_2016	−1.42	−1.64	−1.20
Resting	01_2016	−35.87	−50.43	−21.32
	02_2016	−9.85	−29.10	9.40
	08_2016	−68.23	−85.12	−51.34
	13_2016	−9.50	−25.60	6.61
	17_2016	−36.50	−52.52	−20.48
	19_2016	−19.73	−35.18	−4.28
	21_2016	−2.18	−17.38	13.03
Locomotion	01_2016	−7.25	−21.86	7.36
	02_2016	71.82	52.10	91.55
	08_2016	−32.29	−48.49	−16.09
	13_2016	118.34	102.06	134.62
	17_2016	−87.39	−103.65	−71.14
	19_2016	−6.87	−22.48	8.73
	21_2016	0.60	−14.49	15.68
Balling up	01_2016	94.23	67.43	121.03
	02_2016	−186.25	−222.76	−149.73
	08_2016	96.77	67.07	126.47

	13_2016	−20.82	−50.52	8.88
	17_2016	181.41	151.62	211.20
	19_2016	73.86	45.24	102.49
	21_2016	21.04	−6.62	48.70
other	01_2016	−50.54	−68.05	−33.02
	02_2016	120.74	96.78	144.69
	08_2016	8.34	−11.06	27.74
	13_2016	−88.29	−107.74	−68.84
	17_2016	−56.08	−75.48	−36.67
	19_2016	−47.81	−66.51	−29.11
	21_2016	−21.62	−39.69	−3.55
Diurnality	01_2016	−0.25	−0.34	−0.16
	02_2016	−0.42	−0.51	−0.33
	08_2016	−0.20	−0.29	−0.10
	09_2016	−0.37	−0.50	−0.24
	13_2016	−0.25	−0.34	−0.17
	17_2016	−0.06	−0.16	0.04
	19_2016	0.08	−0.01	0.18
	21_2016	−0.08	−0.17	0.01
Tsdusk	01_2016	0.40	0.28	0.51
	02_2016	0.04	−0.07	0.15
	08_2016	0.87	0.75	0.99
	09_2016	0.09	−0.07	0.25
	13_2016	1.06	0.94	1.17
	17_2016	0.76	0.63	0.89
	19_2016	0.59	0.48	0.70
	21_2016	0.25	0.13	0.36
ODBA	01_2016	−0.23	−0.39	−0.08
	02_2016	0.34	0.13	0.55
	08_2016	−0.60	−0.78	−0.43
	13_2016	0.12	−0.06	0.29
	17_2016	−1.16	−1.33	−0.98
	19_2016	0.55	0.38	0.72
	21_2016	−0.45	−0.61	−0.29
DFC	01_2016	0.01	−0.01	0.03
	02_2016	0.14	0.12	0.16
	08_2016	−0.15	−0.17	−0.13
	09_2016	−0.17	−0.20	−0.13
	13_2016	−0.09	−0.11	−0.07
	17_2016	−0.07	−0.09	−0.05
	19_2016	−0.08	−0.10	−0.06
	21_2016	−0.13	−0.15	−0.10

Table S2. Results for the nightly used area for all individuals.

Animal_ID	Phase	mcp_95	mcp95_ci	kde_95	kde95_ci	kde_50	kde50_ci
01_2016	festival	2.87	0.88	9.10	3.43	2.14	0.85
01_2016	pre-festival	4.20	1.48	14.00	7.25	3.10	1.50
02_2016	festival	2.74	0.69	7.68	1.75	1.93	0.51
02_2016	pre-festival	2.83	0.76	7.84	2.09	2.02	0.55
08_2016	festival	1.34	0.42	4.25	1.65	1.09	0.46
08_2016	pre-festival	2.65	0.47	7.95	1.61	1.99	0.45
09_2016	festival	3.19	1.39	8.43	3.10	2.12	0.81
09_2016	pre-festival	4.98	1.08	13.57	2.73	3.59	0.75
13_2016	festival	1.63	0.32	4.59	0.92	1.04	0.25
13_2016	pre-festival	1.95	0.50	4.57	1.22	1.07	0.29
17_2016	festival	0.93	0.47	2.45	1.26	0.60	0.35

17_2016	pre-festival	2.57	0.56	6.33	1.40	1.46	0.32
19_2016	festival	1.62	0.69	5.02	1.91	1.26	0.51
19_2016	pre-festival	2.95	0.71	11.82	2.84	3.02	0.85
21_2016	festival	4.03	1.25	11.48	3.26	2.92	0.96
21_2016	pre-festival	6.49	1.08	16.93	2.86	4.36	0.82

Shown are mean values of MCP 95%, KDE 95% and KDE 50% for each individual separated by the phase of the festival. CI denoted the 95% confidence interval of the mean areas.

Table 3. Individual nesting data. Every unique nest got a unique id. Repeated usage of the same nest is depicted through the nesttime in days.

Sex	Individual	Nest	Nesttime	Censor	Coding	Research_Interval	Disturbance
m	1	a	5	yes	0	before	no
m	1	b	2	no	1	before	no
m	1	c	11	yes	0	before	no
m	1	c	3	no	1	during	disturb
m	1	d	6	no	1	during	disturb
m	1	e	1	no	1	during	disturb
m	1	f	2	no	1	during	disturb
m	1	g	1	no	1	during	disturb
m	1	h	5	yes	0	during	disturb
m	1	h	1	no	1	after	no
m	1	i	4	yes	0	after	no
f	2	a	5	yes	0	before	no
f	2	b	2	no	1	before	no
f	2	a	2	no	1	before	no
f	2	b	1	no	1	before	no
f	2	a	1	no	1	before	no
f	2	b	1	no	1	before	no
f	2	c	7	yes	0	before	no
f	2	c	8	no	1	during	disturb
f	2	d	1	no	1	during	disturb
f	2	c	1	no	1	during	disturb
f	2	d	1	no	1	during	disturb
f	2	c	2	no	1	during	disturb
f	2	d	5	no	1	during	disturb
f	2	d	5	yes	0	after	no
m	5	a	2	yes	0	before	no
m	5	b	1	no	1	before	no
m	5	a	10	no	1	before	no
m	5	c	4	no	1	before	no
m	5	d	1	no	1	before	no
m	5	e	1	yes	0	before	no
f	7	a	4	yes	0	before	no
f	7	b	1	no	1	before	no
f	7	a	1	no	1	before	no
f	7	b	1	no	1	before	no
f	7	a	1	no	1	before	no
f	7	b	11	yes	0	before	no
f	7	b	10	yes	0	during	disturb
f	7	b	2	yes	0	after	no
f	8	a	16	yes	0	before	no
f	8	b	1	yes	0	before	no
f	8	c	1	no	1	during	disturb

f	8	d	5	no	1	during	disturb
f	8	e	6	no	1	during	disturb
f	8	d	1	no	1	during	disturb
f	8	e	2	no	1	during	disturb
f	8	f	1	no	1	during	disturb
f	8	g	2	yes	0	during	disturb
f	8	g	5	yes	0	after	no
m	9	a	2	yes	0	before	no
m	9	b	1	no	1	before	no
m	9	a	1	no	1	before	no
m	9	b	1	no	1	before	no
m	9	c	1	no	1	before	no
m	9	a	1	no	1	before	no
m	9	c	1	no	1	before	no
m	9	d	1	no	1	before	no
m	9	c	2	yes	0	before	no
m	9	d	1	no	1	before	no
m	9	c	1	no	1	before	no
m	9	d	1	no	1	before	no
m	9	e	1	no	1	before	no
m	9	d	1	yes	0	before	no
m	9	f	1	yes	0	before	no
m	9	f	1	no	1	during	disturb
m	9	d	1	no	1	during	disturb
m	9	f	2	yes	0	during	disturb
m	9	f	3	no	1	during	disturb
m	9	g	1	no	1	during	disturb
m	9	h	6	no	1	during	disturb
m	9	i	2	yes	0	during	disturb
m	9	j	5	yes	0	after	no
m	10	a	1	yes	0	before	no
m	10	b	1	yes	0	before	no
m	10	a	1	yes	0	before	no
m	10	c	5	yes	0	before	no
m	11	a	5	yes	0	before	no
m	11	b	2	no	1	before	no
m	11	a	1	no	1	before	no
m	11	c	3	no	1	before	no
m	11	b	1	no	1	before	no
m	11	c	1	no	1	before	no
m	11	a	1	no	1	before	no
m	11	c	4	no	1	before	no
m	11	d	1	yes	0	before	no
m	11	c	3	no	1	during	disturb
m	11	d	1	no	1	during	disturb
m	11	c	1	no	1	during	disturb
m	11	e	1	no	1	during	disturb
m	11	d	1	no	1	during	disturb
m	11	a	1	no	1	during	disturb
m	11	e	2	no	1	during	disturb
m	11	d	1	no	1	during	disturb
m	11	e	2	no	1	during	disturb
m	11	d	1	no	1	during	disturb
f	13	a	1	yes	0	before	no
f	13	b	1	no	1	before	no
f	13	c	2	no	1	before	no

f	13	d	7	no	1	before	no
f	13	c	1	no	1	before	no
f	13	e	2	no	1	before	no
f	13	d	1	no	1	before	no
f	13	f	1	no	1	before	no
f	13	e	2	no	1	before	no
f	13	d	1	yes	0	before	no
f	13	d	1	no	1	during	disturb
f	13	e	1	no	1	during	disturb
f	13	d	1	no	1	during	disturb
f	13	g	2	no	1	during	disturb
f	13	f	8	no	1	during	disturb
f	13	h	5	yes	0	during	disturb
f	13	i	5	yes	0	after	no
m	14	a	2	yes	0	before	no
m	14	b	4	no	1	before	no
m	14	a	3	no	1	before	no
m	14	b	2	no	1	before	no
m	14	a	1	no	1	before	no
m	14	b	1	no	1	before	no
m	14	c	1	no	1	before	no
m	14	b	1	no	1	before	no
m	14	d	1	no	1	before	no
m	14	b	3	yes	0	before	no
m	14	d	1	no	1	during	disturb
m	14	e	7	no	1	during	disturb
m	14	f	1	no	1	during	disturb
m	14	e	1	no	1	during	disturb
m	14	f	1	no	1	during	disturb
m	14	g	1	no	1	during	disturb
m	14	f	1	no	1	during	disturb
m	14	g	1	no	1	during	disturb
m	14	h	1	no	1	during	disturb
m	14	g	3	yes	0	during	disturb
m	14	g	5	yes	0	after	no
f	16	a	1	yes	0	before	no
f	16	b	2	no	1	before	no
f	16	a	1	no	1	before	no
f	16	c	1	no	1	before	no
f	16	d	1	no	1	before	no
f	16	c	1	no	1	before	no
f	16	a	1	no	1	before	no
f	16	d	2	no	1	before	no
f	16	a	1	no	1	before	no
f	16	d	7	no	1	before	no
f	16	e	1	yes	0	before	no
f	16	d	1	no	1	during	disturb
f	16	e	3	no	1	during	disturb
f	16	d	9	no	1	during	disturb
f	16	f	5	yes	0	during	disturb
f	16	f	5	yes	0	after	no
f	17	a	1	yes	0	before	no
f	17	b	1	no	1	before	no
f	17	a	2	no	1	before	no
f	17	b	10	no	1	before	no
f	17	c	3	no	1	before	no

f	17	d	1	no	1	before	no
f	17	e	1	yes	0	before	no
f	17	e	10	no	1	before	disturb
f	17	f	5	no	1	before	disturb
f	17	c	3	yes	0	during	disturb
f	17	c	2	no	1	after	no
f	17	e	2	no	1	after	no
f	17		1	yes	0	after	no
m	18	a	18	yes	0	before	no
m	18	b	1	yes	0	before	no
m	18	c	4	no	1	during	disturb
m	18	d	8	no	1	during	disturb
m	18	e	6	yes	0	during	disturb
m	18	e	5	yes	0	after	no
m	19	a	1	yes	0	before	no
m	19	b	2	no	1	before	no
m	19	a	1	no	1	before	no
m	19	c	1	no	1	before	no
m	19	b	1	no	1	before	no
m	19	d	5	no	1	before	no
m	19	c	1	no	1	before	no
m	19	d	2	no	1	before	no
m	19	e	1	no	1	before	no
m	19	d	4	yes	0	before	no
m	19	d	3	no	1	during	disturb
m	19	f	3	no	1	during	disturb
m	19	g	3	no	1	during	disturb
m	19	h	6	no	1	during	disturb
m	19	g	3	yes	0	during	disturb
m	19	g	3	no	1	after	no
m	19	i	2	yes	0	after	no
f	20	a	1	yes	0	before	no
f	20	b	3	no	1	before	no
f	20	c	8	no	1	before	no
f	20	d	18	yes	0	during	disturb
f	20	d	5	yes	0	after	no
m	21	a	3	yes	0	before	no
m	21	b	1	no	1	before	no
m	21	c	1	no	1	before	no
m	21	b	8	no	1	before	no
m	21	d	1	yes	0	before	no
m	21	b	1	no	1	before	no
m	21	c	1	no	1	before	no
m	21	d	1	yes	0	before	no
m	21	b	1	no	1	during	disturb
m	21	c	1	no	1	during	disturb
m	21	b	1	no	1	during	disturb
m	21	d	2	no	1	during	disturb
m	21	b	1	no	1	during	disturb
m	21	e	1	no	1	during	disturb
m	21	f	1	no	1	during	disturb
m	21	e	3	no	1	during	disturb
m	21	b	2	no	1	during	disturb
m	21	g	1	no	1	during	disturb
m	21	e	1	no	1	during	disturb
m	21	c	1	no	1	during	disturb

m	21	g	1	no	1	during	disturb
m	21	h	1	yes	0	during	disturb
m	21	h	3	no	1	after	no
m	21	d	2	yes	0	after	no
m	22	a	1	yes	0	before	no
m	22	b	2	no	1	before	no
m	22	c	2	no	1	before	no
m	22	d	1	no	1	before	no
m	22	c	1	no	1	before	no
m	22	d	2	no	1	before	no
m	22	c	1	no	1	before	no
m	22	d	1	no	1	before	no
m	22	c	1	yes	0	before	no
m	22	d	1	no	1	during	disturb
m	22	a	4	no	1	during	disturb
m	22	e	1	no	1	during	disturb
m	22	f	3	no	1	during	disturb
m	22	g	2	no	1	during	disturb
m	22	h	1	no	1	during	disturb
m	22	i	2	no	1	during	disturb
m	22	h	1	no	1	during	disturb
m	22	j	1	no	1	during	disturb
m	22	h	1	no	1	during	disturb
m	22	g	1	yes	0	during	disturb
m	22	h	1	no	1	after	no
m	22	g	2	no	1	after	no
m	22	h	1	no	1	after	no
m	22	g	1	yes	0	after	no
m	23	a	2	yes	0	after	no
f	24	a	9	no	1	during	disturb
f	24	b	1	no	1	during	disturb
f	24	a	5	no	1	during	disturb
f	24	b	3	yes	0	during	disturb
f	24	b	5	yes	0	after	no

Table 4. The data from which the SVM model was built.

Animal ID	Number of Days Removed total(pre-festival/festival)	Dates
01_2016	6 (3/3)	2016.08.15, 2016.08.23, 2016.08.25, 2016.09.01, 2016.09.06, 2016.09.08
02_2016	14 (9/5)	2016.08.10, 2016.08.15, 2016.08.22, 2016.08.23, 2016.08.25, 2016.09.01, 2016.09.06, 2016.09.07, 2016.09.08, 2016.09.13
08_2016	9 (3/6)	2016.08.15, 2016.08.23, 2016.08.25, 2016.09.01, 2016.09.06, 2016.09.07, 2016.09.08, 2016.09.13, 2016.09.14
09_2016	16 (3/13)	2016.08.16, 2016.08.22, 2016.08.25, 2016.09.01, 2016.09.03, 2016.09.04, 2016.09.09, 2016.09.12, 2019.09.16
13_2016	9 (3/6)	2016.08.15, 2016.08.16, 2016.08.25, 2016.09.01, 2016.09.04, 2016.09.06, 2016.09.07, 2016.09.08, 2016.09.13
17_2016	10 (3/7)	2016.08.16, 2016.08.17, 2016.08.25, 2016.09.01, 2016.09.06, 2016.09.08, 2016.09.13, 2016.09.14
19_2016	8 (3/5)	2016.08.16, 2016.08.22, 2016.08.23, 2016.08.29, 2016.09.01, 2016.09.07, 2016.09.08, 2016.09.13
21_2016	7 (3/4)	2016.08.16, 2016.08.23, 2016.08.25, 2016.09.01, 2016.09.06, 2016.09.08, 2016.09.13

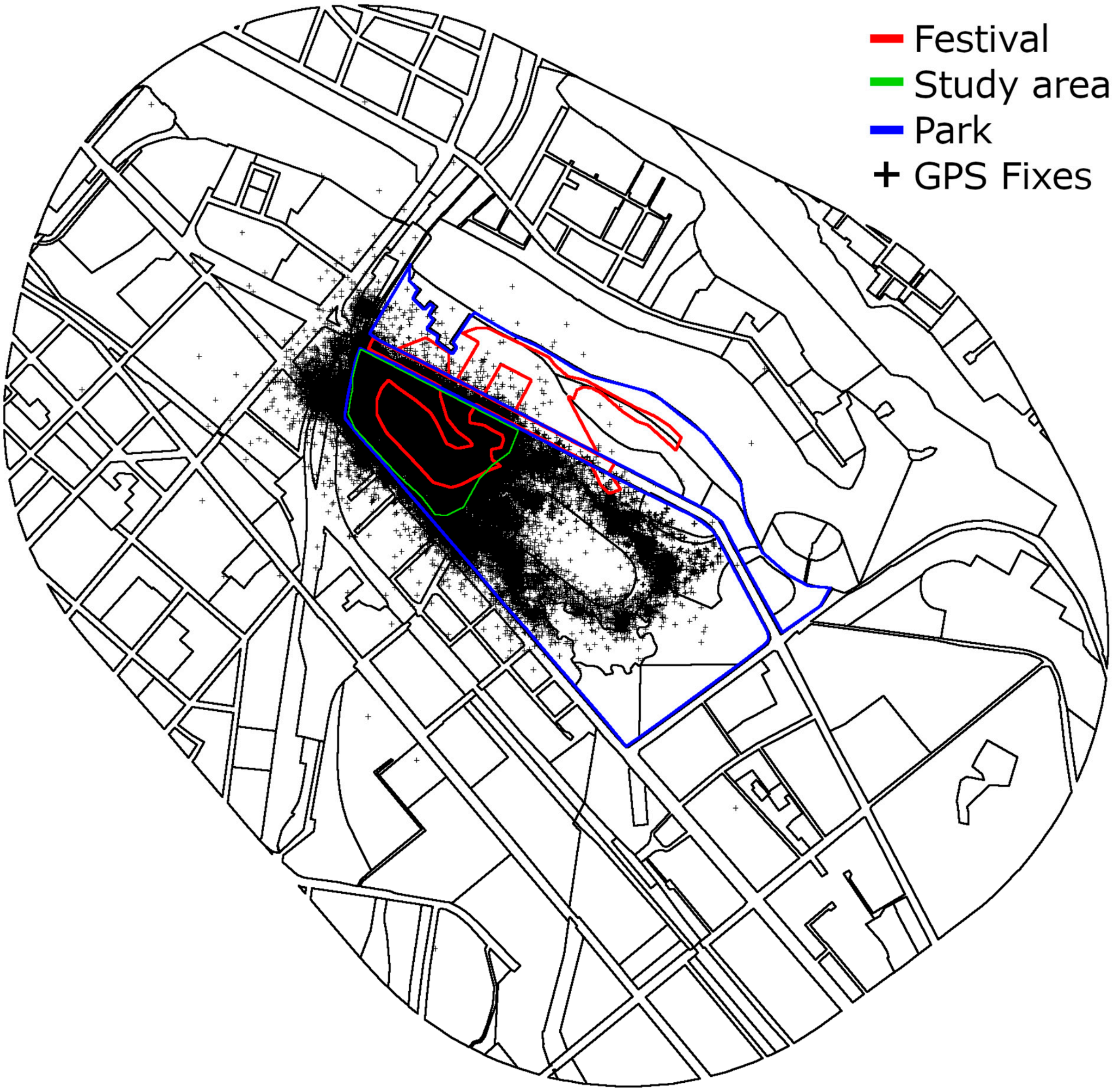

Figure S1. Map of the Park with surrounding area. The Park Area is indicated by the blue lines, the festival area is indicated by the red lines and the study area is indicated by the green line. GPS fixes are shown for all study hedgehogs. Supplementary Figures 3 and 4 show GPS fixes for only the pre-festival phase and the festival phase respectively. The majority of fixes are located in the study area. More than half of the study area was used for the festival. The study hedgehogs leave the park on a few occasions.

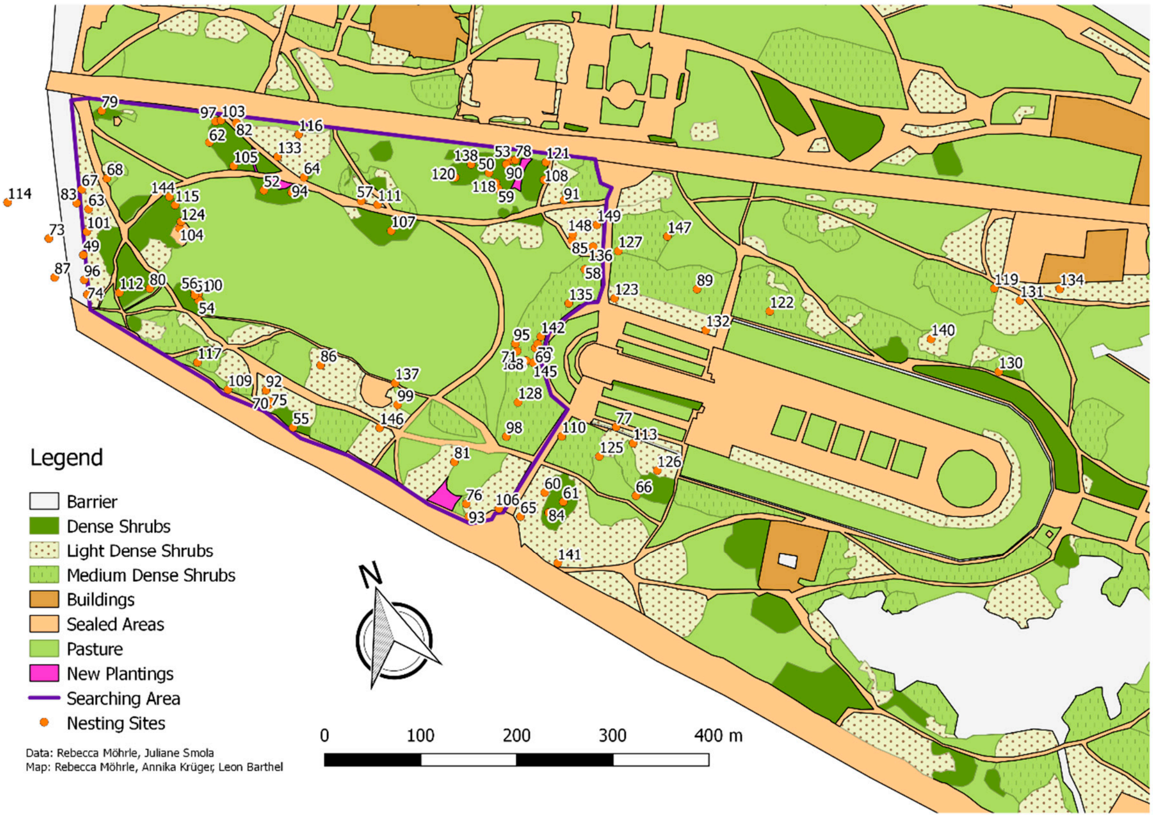
Legend
Barrier
Dense Shrubs
Light Dense Shrubs
Medium Dense Shrubs
Buildings
Sealed Areas
Pasture
New Plantings
Searching Area
Nesting Sites
Data: Rebecca Möhrle, Juliane Smola
Map: Rebecca Möhrle, Annika Krüger, Leon Barthel
N
0
100
200
300
400 m

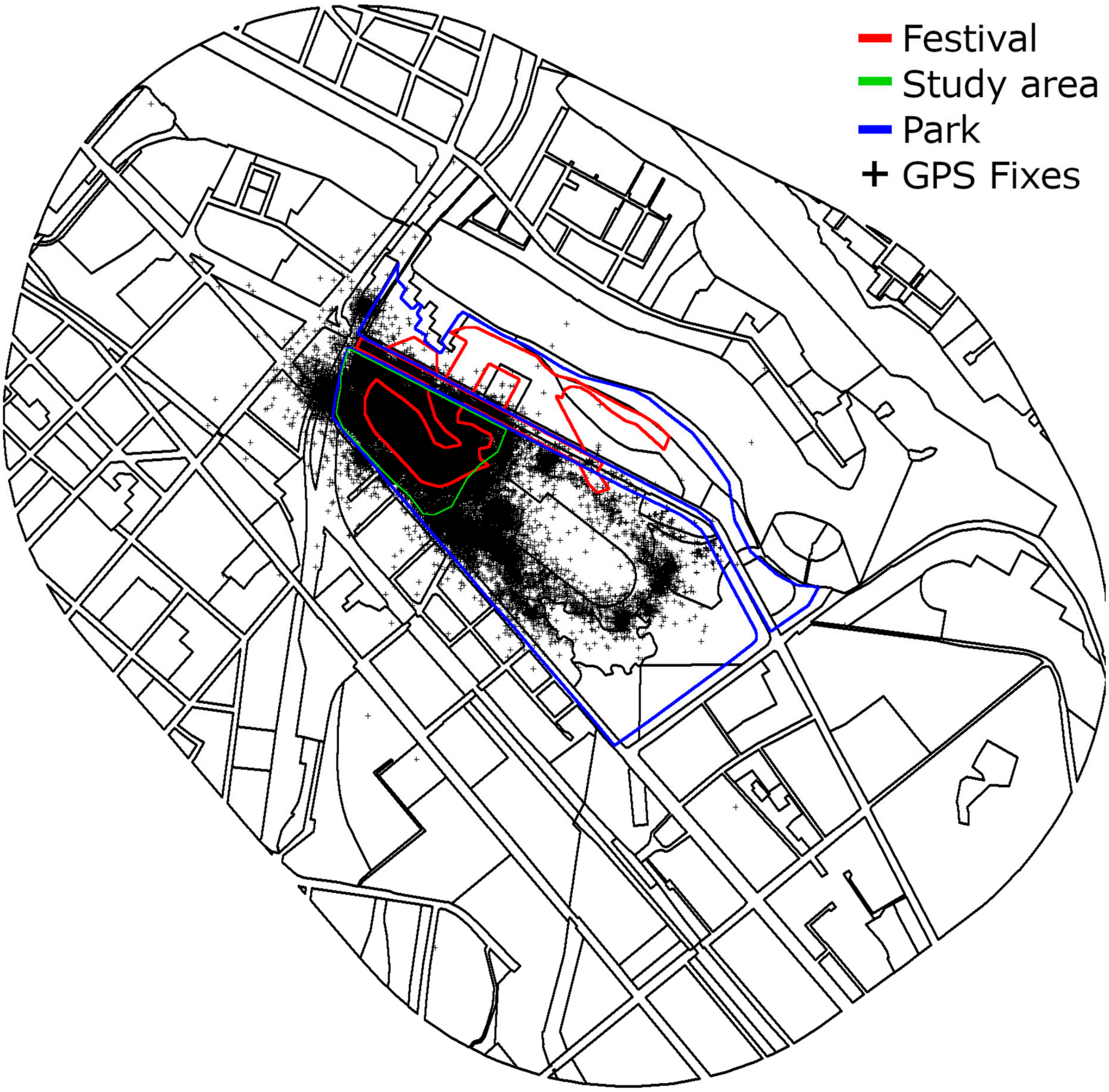

Figure S2. Map of the Park with surrounding area before the festival. The Park Area is indicated by the blue lines, the festival area is indicated by the red lines and the study area is indicated by the green line. GPS fixes are shown for all study hedgehogs. The majority of fixes are located in the study area. More than half of the study area was used for the festival. The study hedgehogs leave the park on a few occasions.

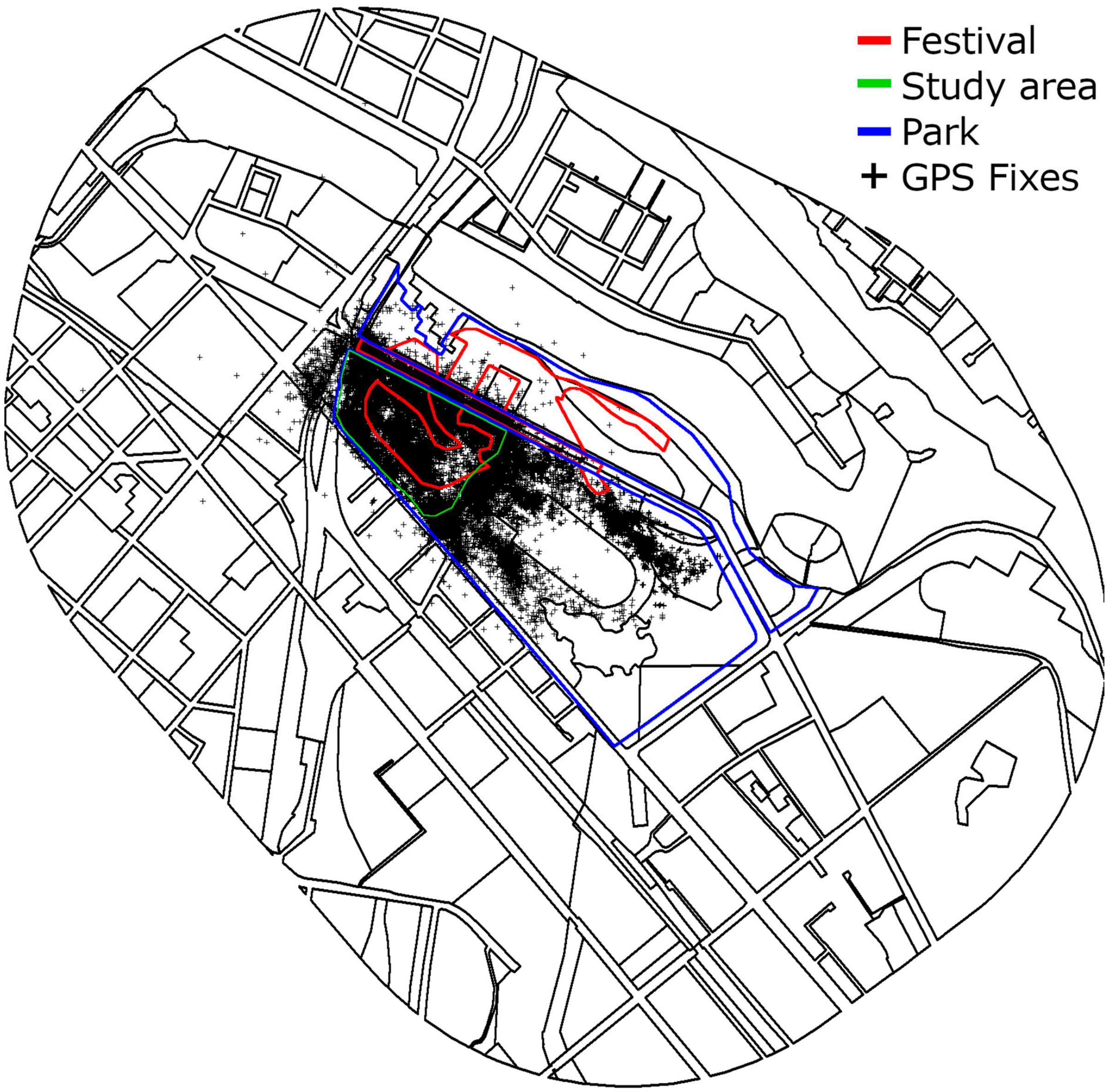

Figure S3. Map of the Park with surrounding area during the festival. The Park Area is indicated by the blue lines, the festival area is indicated by the red lines and the study area is indicated by the green line. GPS fixes are shown for all study hedgehogs. The majority of fixes are located in the study area. More than half of the study area was used for the festival. The study hedgehogs leave the park on a few occasions.

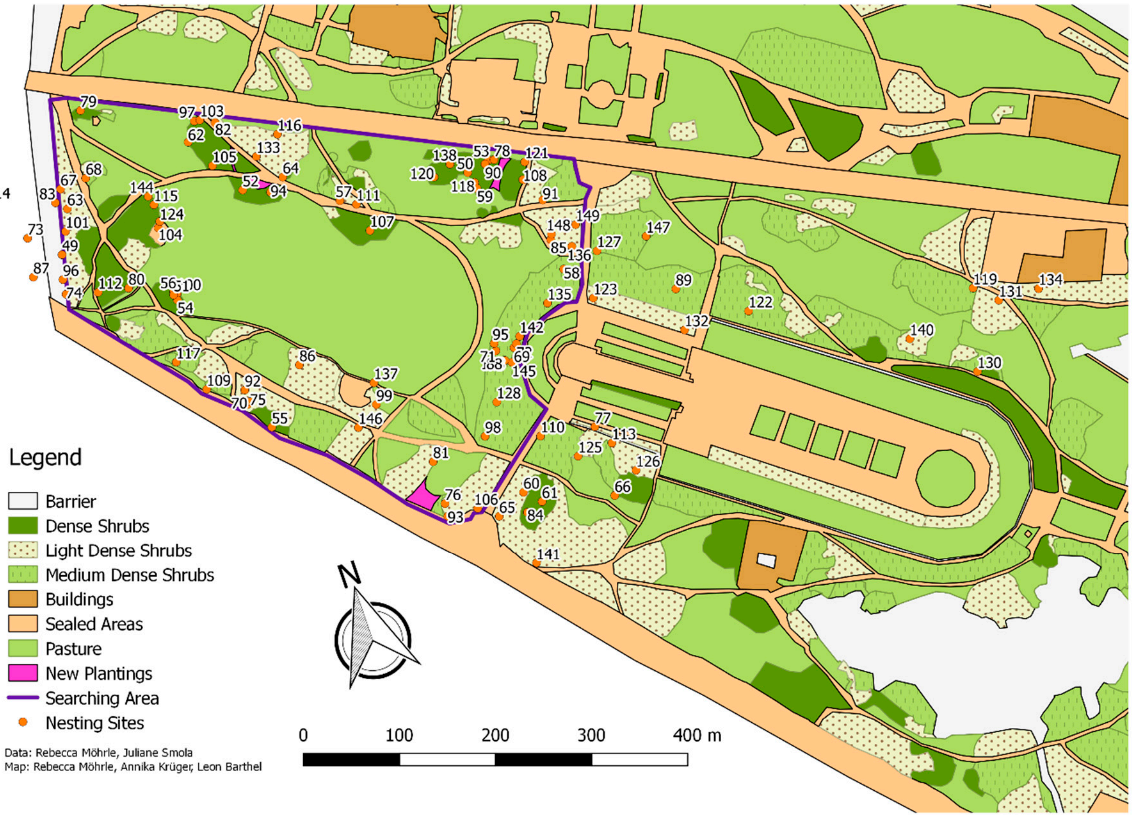

Figure S4. Map of all nest locations. Nests are restricted to areas covered by bushes. The majority of bushes are within the study area. Search area corresponds to the study area in Figures S1-S3.
